# Role of *NCAN* rs2228603 polymorphism in the incidence of nonalcoholic fatty liver disease: a case-control study

**DOI:** 10.1186/s12944-016-0367-4

**Published:** 2016-11-26

**Authors:** Meng-Juan Wu, Chen Yuan, Lin-Lin Lu, Bai-Quan An, Shi-Ying Xuan, Yong-Ning Xin

**Affiliations:** 1 Department of Gastroenterology, Qingdao Municipal Hospital, Nanjing Medical University, Qingdao, 266071 Shandong Province China; 2 Digestive Disease Key Laboratory of Qingdao, Qingdao, Shandong Province China; 3 Central Laboratories, Qingdao Municipal Hospital, Qingdao, Shandong Province China; 4 Department of Gastroenterology, Qingdao Municipal Hospital, Qingdao, Shandong Province China

**Keywords:** *NCAN*, rs2228603, Polymorphism, Non-alcoholic fatty liver disease

## Abstract

**Background:**

Recently genome-wide association studies identified that *NCAN* rs2228603 polymorphism was associated with non-alcoholic fatty liver disease (NAFLD) mainly in subjects of European ancestry. While no research have been conducted to demonstrate the relationship between *NCAN* rs2228603 and NAFLD in Chinese Han adults. The aim of this study was to investigate whether *NCAN* rs2228603 is associated with NAFLD in Chinese population.

**Methods:**

Gene *NCAN* rs2228603 was genotyped in 182 patients with NAFLD and 195 healthy controls. The expression of *NCAN* was tested according to polymerase chain reaction analysis (PCR) and serum lipids were performed by biology techniques.

**Results:**

No significant difference was found in genotype and allele frequencies of *NCAN* rs2228603 between the NAFLD group and the controls (*P* > 0.05). Subjects with the *NCAN* rs2228603 CT genotype showed a higher level of alkaline phosphatase (AKP) (*P* = 0.017) and a higher high-density lipoprotein (HDL) (*P* < 0.05).

**Conclusions:**

Our study for the first time identified that the gene *NCAN* rs2228603 is not a risk factor for the incidence of NAFLD in Chinese population. Also we found the dual and opposite role of T variant in protecting liver with a higher level of HDL and conferring risk for liver damage with a higher level of AKP.

**Trial registration:**

Chinese Clinical Trial Register.gov Identifier: ChiCTR-ROC-15006447.

## Background

Non-alcoholic fatty liver disease (NAFLD), a hepatic manifestation of metabolic syndrome, is an emerging public health concern which is prevalent in individuals with metabolic derangements including obesity, insulin resistance, and diabetes mellitus [1, 2]. As a common chronic liver disease, its spectrum ranged from simple steatosis, non-alcoholic steatohepatitis (NASH), fibrosis/cirrhosis and hepatocellular carcinoma [3, 4]. NAFLD is predicted to be the main course for liver disease world-wide by 2020 [5]. Epidemiologic studies showed a prevalence of about 20~30% in Western countries [6, 7] and 12~15% in China depending on population and investigative methods [8], which was similar to some previous studies [9–11]. NAFLD prevalence was confirmed by variety of tools, which led to the difference. Moreover, In some areas of China where obesity is more common, which will continue to grow in a setting of increasing rates of NAFLD and other metabolic syndrome [12]. Recently, patients with NAFLD exhibited a high and rising mortality rate [13, 14]. The most frequent causes of death are represented by liver-related diseases, cardiovascular disease and malignantneoplasms [15]. It is considered that the main course of the death in patients with NAFLD is from coronary events [16–18]. Cardiovascular disease accounted for about 25% deaths in patients with NAFLD [19, 20]. The pathogenesis of NAFLD is still a complex disorder and multifaceted process which remains under investigation. Host genetics are considered as the significant factor for the formation and development of NAFLD [21]. Genome-wide association studies had assessed the correlation between *NCAN* and NAFLD among different ethnic groups [14, 22].

*NCAN* contains at least 20 genes in a 500 kb region on chromosome 19p13 and expresses neurocan, a chondroitin sulphate proteoglycan that thought to be involved in celladhesion and migration in the nervous system [23–26]. Interestingly, Nischalke’s study showed *NCAN* is not only expressed in neuronal tissue, but also in the liver [27–29]. Studies found that the SNP rs2228603 in the *NCAN* gene, resulting in an amino acid exchange (proline to serine) at position 92, was strongly related to the plasma low-density lipoprotein (LDL) and triglyceride (TG) levels [26]. *N* *CAN* is used to be recognized just that the central nervous system (CNS) is an important regulator of peripheral glucose and triglyceride metabolism. More studies have demonstrated that SNP rs2228603 was associated with hepatic steatosis [23, 30]. However, *NCAN* rs2228603 has been involved in a few studies on NAFLD among Asian. A recent study showed that SNP in *NCAN* was related to the higher level of ALT in Indian subjects [31]. However, Lin et al. studies showed *NCAN* was not a risk factor for NAFLD in obese Taiwanese children [32]. There is no research conducted between the polymorphism of *NCAN* and NAFLD in Chinese Han adults. The aim of this study was to investigate whether *NCAN* rs2228603 is associated with NAFLD in Chinese population.

## Methods

### Subjects

This study was performed in accordance with the principles of declaration of Helsinki and its appendices [33] and approved by the ethical committee of Qingdao municipal hospital (Qingdao, China). All patients had provided written informed consent before participation in the study.

We selected a total of 377 unrelated adult subjects from August 2012 to August 2015, including 182 patients of different genders and different ages (85 males, 97 females,) diagnosed with NAFLD and 195 healthy controls matched for genders and ages (88 males, 107 females,) who underwent B-type ultrasonography. We collected subjects from the department of gastroenterology and the medical center of Qingdao municipal hospital. All individuals were unrelated and ethnically Han Chinese adults. The diagnosis of NAFLD was made by ultrasonic imaging according to EASL and AASLD criteria. Other causes of liver disease were excluded, including increased alcohol intake (>210/140 g/week for males/females), as confirmed by at least one family member or friend and carboxydesialylated transferrin determination, viral and autoimmune hepatitis, hereditary hemochromatosis, andalphal-antitrypsin deficiency [11]. The healthy controls were confirmed using general laboratory examinations and medical examinations at the same hospital.

### Biochemical parameters

We collected venous blood samples after a 12-h overnight fast from all participants. The following data for each subject was obtained: height, body weight, waist circumference and hip circumference. Total cholesterol (TC), Triglyceride (TG), high-density lipoprotein (HDL) and low-density lipoprotein (LDL) were measured by routine enzymatic methods. Serum concentrations of gamma-glutamyltranspeptidase (GGT), Total bilirubin (TBIL), alkaline phosphatase (AKP), glucose (GLU), Uric acid (UA), alanine aminotransferase (ALT) and aspartate aminotransferase (AST) were tested with available standardized methods. Environmental factors were excluded in the study.

### Genotyping

Genomic DNA was isolated from peripheral blood using purification kit (BioTeke, Biotechnology, Beijing, China) according to the manufacturer’s instructions. After extraction, the genomic DNA was stored at −20 °C before use. Genotyping for *NCAN* (rs2228603) was performed by polymerase chain reaction (PCR) using the following primers for *NCAN* polymorphism: 5′-TGGCATCGTGATGGACTCC-3′, 5′-AATGTCACGCACGATTTCCC-3′. PCR amplification was performed under the following conditions: 10 min at 95 °C, then 50 cycles before denaturation at 94 °C for 1 min, annealing at 60 °C for 1 min and elongation 1 min at 70 °C. Direct DNA sequencing, the ABI Prism sequence detection system ABI3730 (Foster city, CA, USA), was taken for the assay of *NCAN* genotypes. The average genotype call rate was >95% and the genotype concordance rate of blind replicates was >99%.

### Statistical analysis

Statistical analysis was carried out using SPSS Statistics software version 17.0 (SPSS Inc. Chicago, IL, USA). Genotype and alleles were obtained using chi-square test and the *χ*2 test was applied to assess DNA distributions between NAFLD patients and the controls. The baseline characteristics of participants were presented as mean ± standard deviation. The differences in characteristics between different groups were tested by the Student’s *t* test, paired samples *t* test or *χ*2 test. Logistic regression analysis was performed to estimate the association of polymorphism with NAFLD. A *P* value < 0.05 was considered statistically significant.

## Results

### Clinical characteristics of the participants

We investigated 182 NAFLD patients and 195 controls matched for age (*P* = 0.000) and gender (*P* = 0.759). Table 1 showed the clinical characteristics and serum lipid levels of the subjects. Compared to the controls, NAFLD patients showed increased age, weight, Waist circumference, Hip circumference, serum ALT, AST, GGT, AKP, GLU, UA, TG, TC and LDL, however the level of HDL decreased. These results had significant differences.

**Table 1:** Demographics and clinical characteristics of Patients with NAFLD and Controls^a^

Characteristics	NAFLD patients (*n* = 182)	Controls (*n* = 195)	*p* Value
Age, y	46.09 ± 11.59	40.64 ± 11.53	0.000
Gender, Female/Male	97/85	107/88	0.759
Height, cm	167.17 ± 10.55	166.09 ± 6.46	0.237
Weight, kg	74.28 ± 11.44	63.38 ± 11.10	0.000
Waist circumference, cm	92.29 ± 9.29	82.46 ± 8.85	0.000
Hip circumference, cm	103.11 ± 8.21	96.87 ± 8.80	0.000
ALT, U/L	25.63 ± 14.41	19.71 ± 10.49	0.000
AST, U/L	21.90 ± 11.23	19.64 ± 6.46	0.018
GGT, U/L	22.19 ± 9.62	14.88 ± 5.69	0.000
TBIL, umol/L	13.95 ± 6.40	13.36 ± 5.30	0.329
AKP, U/L	68.18 ± 18.35	59.79 ± 13.81	0.000
GLU, mmol/L	5.57 ± 1.79	4.96 ± 1.20	0.000
UA, umol/L	364.42 ± 187.65	301.10 ± 76.82	0.000
TG, mmol/L	1.98 ± 1.78	1.27 ± 1.58	0.000
TC, mmol/L	4.92 ± 0.95	4.64 ± 0.93	0.004
HDL, mmol/L	1.29 ± 0.46	1.48 ± 0.33	0.000
LDL, mmol/L	3.30 ± 0.93	2.94 ± 0.80	0.000

*Abbreviation*: *ALT* Alanine aminotransferase, *AST* Aspartate aminotransferase, *GGT* gamma-glutamyltranspeptidase, *TBIL* Total bilirubin, *AKP* alkaline phosphatase, *GLU* glucose, *UA* Uric acid, *TG* Triglyceride, *TC* Total cholesterol, *HDL* high-density lipoprotein, *LDL* low-density lipoprotein

^a^Data are presented as Mean ± SD

### NCAN rs2228603 genotypes and allele distribution

The genotypes distribution of the *NCAN* rs2228603 corresponded to the Hardy-Weinberg equilibriumin in NAFLD and control groups (P_NAFLD_ =0.179; P_control_ =0.101, respectively). To ensure the accuracy of genotyping, DNA sequencing was repeated in 150 subjects for reverse sequencing. Distribution of *NCAN* genotypes was shown in Table 2, which demonstrated that there were no significant differences between the patients with NAFLD and the healthy controls (*P* > 0.05). The gene *NCAN* rs2228603 was not a risk factor in the prevalence and pathogenesis of NAFLD (OR = 0.832, 95% CI: 0.499–1.386).

**Table 2:** Correlation of the polymorphism in gene *NCAN* with risk of NAFLD^a^

NCAN (Rs2228603)	NAFLD patients (*n*)	Controls (*n*)	*χ* ^2^	*P* value
Genotypes			0.500	0.480
CT	33	41		
CC	149	154		
TT	0	0		
Allele C			0.445	0.505
T	33	41		
C	331	349		

*Abbreviation*: *NAFLD* non-alcoholic fatty liver disease patients

^a^ *p*: NAFLD patients vs. Control

### Genetic association of NCAN rs2228603 with NAFLD

To evaluate whether the selected SNP influence the laboratory parameters, all variants in carriers and non-carriers were examined. As shown in Table 3, there was strongly difference in AKP (*P* = 0.017) between subjects with allele T and subjects without allele T in overall series. Meanwhile, a significant difference of HDL (*P* < 0.05) was observed in all subjects and healthy controls. However, other serological markers did not reach the statistical difference (*P* > 0.05).

**Table 3:** Analysis of clinical characteristics in *NCAN* (*rs2228603* C/T) carriers and non-carriers of the study population^a^

Characteristic	Overall series			NAFLD patients			Controls		
	Carriers	Non-carriers	*P*	Carriers	Non-carriers	*P*	Carriers	Non-carriers	*P*
Age, y	44.74 ± 11.20	42.91 ± 12.01	0.234	48.58 ± 10.69	45.54 ± 11.74	0.174	41.66 ± 10.74	40.36 ± 11.75	0.524
Female/male	45/29	200/103	0.401	14/19	83/66	0.166	31/10	117/37	0.961
Height, cm	167.35 ± 7.76	166.43 ± 8.89	0.416	168.45 ± 8.20	166.89 ± 11.00	0.441	166.46 ± 7.36	166.00 ± 6.22	0.682
Weight, Kg	68.41 ± 11.77	68.70 ± 12.67	0.857	71.39 ± 10.34	74.91 ± 11.61	0.110	66.00 ± 12.41	62.69 ± 10.60	0.123
ALT, U/L	22.43 ± 12.41	22.60 ± 12.99	0.920	24.55 ± 12.09	25.87 ± 14.90	0.633	20.73 ± 12.55	19.44 ± 9.90	0.483
AST, U/L	20.68 ± 8.04	20.75 ± 9.40	0.953	20.45 ± 5.84	22.22 ± 12.09	0.415	20.85 ± 9.52	19.32 ± 5.36	0.177
GGT, U/L	17.77 ± 7.631	18.56 ± 8.872	0.479	20.67 ± 8.855	22.52 ± 9.776	0.317	15.44 ± 5.572	14.73 ± 5.735	0.482
TBIL, umol/L	13.43 ± 5.41	13.70 ± 5.97	0.725	12.28 ± 4.94	14.32 ± 6.64	0.097	14.36 ± 5.64	13.09 ± 5.19	0.174
AKP, U/L	65.18 ± 13.75	64.73 ± 17.22	0.017	69.94 ± 16.51	69.34 ± 18.60	0.070	67.95 ± 10.75	60.28 ± 14.50	0.258
Glu. mmol/L	5.04 ± 0.86	5.31 ± 1.66	0.182	5.21 ± 1.09	5.66 ± 1.90	0.194	4.91 ± 0.60	4.98 ± 1.32	0.756
UA, umol/L	307.59 ± 81.35	337.55 ± 156.11	0.111	335.42 ± 98.44	370.85 ± 201.84	0.328	285.20 ± 56.33	305.33 ± 81.03	0.136
TG, mmol/L	1.46 ± 1.28	1.65 ± 1.81	0.381	2.00 ± 1.67	1.98 ± 1.81	0.964	1.03 ± 0.57	1.34 ± 1.75	0.263
TC, mmol/L	4.80 ± 0.92	4.77 ± 0.96	0.825	4.88 ± 0.83	4.93 ± 0.98	0.799	4.73 ± 0.98	4.62 ± 0.92	0.493
HDL, mmol/L	1.47 ± 0.25	1.37 ± 0.44	0.009	1.31 ± 0.26	1.29 ± 0.50	0.861	1.60 ± 0.14	1.44 ± 0.36	0.000
LDL, mmol/L	3.04 ± 0.85	3.13 ± 0.89	0.452	3.18 ± 0.68	3.33 ± 0.98	0.403	2.94 ± 0.96	2.94 ± 0.76	0.985

*Abbreviations*: *ALT* Alanine aminotransferase, *AST* Aspartate aminotransferase, *GGT* gamma-glutamyltranspeptidase, *TBIL* Total bilirubin, *AKP* alkaline phosphatase, *GLU* glucose, *UA* Uric acid, *TG* Triglyceride, *TC* Total cholesterol, *HDL* high-density lipoprotein, *LDL* low-density lipoprotein

^a^Data are presented as Mean ± SD

## Discussion

Recently the gene *NCAN* on NAFLD has been studied, however, the results were controversial [23, 34]. The present study firstly evaluated the association between rs2228603 polymorphism in the gene *NCAN* and NAFLD in Chinese population. Our study selected 182 NAFLD patients and 195 healthy controls to observe the correlation between *NCAN* rs2228603 and NAFLD. We found no carriers with TT genotype both in NAFLD and control group, also no significant association of *NCAN* rs2228603 with NAFLD was observed in our subjects, which latter was consistent with some previous findings [32, 34, 35]. While Speliotes et al. identified that variant in the gene *NCAN* was strongly associated with histologic NAFLD and increasing computed tomography (CT) hepatic steatosis [14]. Rs2228603 is located in exon 3 of *NCAN* and encodes anon-conservative nonsynonymous mutation (Pro92Ser), which is predicted by the software tool PolyPhen-2 to alter protein structure and function [36]. It is plausible that this variant increases the risk for NAFLD through influencing dietary fat intake by modulated by a brain-liver axis [26, 37]. We characterized the effect of allele T variant in our groups, which expand further evidence for central control of liver lipid metabolism.

We found, for the first time, an obvious difference between variant carriers and non-carriers regarding AKP. The results showed that *NCAN* rs2228603 CT genotype had a higher level of AKP in overall subjects. AKP is a hydrolase enzyme that commonly used as signal amplification in bone metabolism. Matthew’s study showed that isolated elevated AKP and risk factors for NAFLD should be evaluated for evidence of significant steatohepatitis [38]. However, the mechanism of AKP in the pathogenesis of NAFLD remains unclear and warrants further exploration. The result may provide evidence that carriage of T allele can elevate AKP to strengthen liver damage.

Meanwhile, we found a protected role of the variant in lipid metabolism, and observed that the T allele carriers had a higher serum HDL level than the T allele non-carriers, whereas no significant correlation was observed in LDL-cholesterol, total cholesterol and triglyceride levels with variant carriers. Gorden et al. study found T allele carriers showed a lower serum LDL-cholesterol, total cholesterol and triglyceride levels, particularly in those with NAFLD [23]. Approximately 20~80% NAFLD patients have dyslipidemia [39]. HDL is negatively related to the incidence of atherosclerosis and a decreased level of HDL might indicate the clinical damage to liver cells, while we could not deny the possible influence of the elevated HDL-cholesterol on the association between *NCAN* and NAFLD among different population. A recent study showed that *NCAN* is expressed at the luminal side of cholangiocytes and in vesicles of hepatocytes, which may imply that besides its role in the nervous system, *NCAN* may be involved in transport processes of the liver [27]. Elevated serum HDL-cholesterol has certain protective effect to the liver; however, the mechanism is still unclear and further studies are needed to validate this argument.

## Conclusion

In conclusion, this study elucidated that there was no association between *NCAN* rs2228603 polymorphism and incidence of NAFLD in Chinese population for the first time. The *NCAN* rs2228603 T-allele significantly affects serum AKP and HDL. We can assume that *NCAN* rs2228603 is mainly involved in the progress of NASH by influence liver damage based on simple steatosis. The variant may be a protective factor in early stage. Further studies with large scale of subjects and different ethnicity are needed to estimate the impact of *NCAN* rs2228603 on NAFLD. Hopefully, additional research on the role of *NCAN* in NAFLD might help to elevate the application of future therapeutic strategies and interventions.
